# Assessing causal relationships between sarcopenia and nonalcoholic fatty liver disease: A bidirectional Mendelian randomization study

**DOI:** 10.3389/fnut.2022.971913

**Published:** 2022-11-09

**Authors:** Ze-Hua Zhao, Juanjuan Zou, Xin Huang, Yu-Chen Fan, Kai Wang

**Affiliations:** ^1^Department of Hepatology, Qilu Hospital of Shandong University, Jinan, China; ^2^Department of Otorhinolaryngology, Qilu Hospital of Shandong University, National Health Commission (NHC) Key Laboratory of Otorhinolaryngology (Shandong University), Jinan, China; ^3^Division of Bariatric and Metabolic Surgery, Department of General Surgery, Qilu Hospital of Shandong University, Jinan, China; ^4^Institute of Hepatology, Shandong University, Jinan, China

**Keywords:** sarcopenia, nonalcoholic fatty liver disease—NAFLD, Mendelian randomization (MR), lean body mass, causality

## Abstract

**Background and aims:**

Sarcopenia has been demonstrated to be closely associated with nonalcoholic fatty liver disease (NAFLD). However, whether there are causal relationships between sarcopenia and NAFLD remains undetermined. Here, we aim to address the question using a two-sample bidirectional Mendelian randomization (MR) analysis approach.

**Methods:**

We performed a two-sample bidirectional MR study using summary-level data from genome-wide association studies (GWAS) of the whole body lean mass (*n* = 38,292), appendicular (arms and legs) lean mass (*n* = 28,330), and NAFLD (1,483 biopsy-proven NAFLD cases and 17,781 controls). We first conducted MR analysis with five single nucleotide polymorphisms (SNPs) as genetic instruments for whole body lean mass and three SNPs as instruments for appendicular lean mass to estimate the causal effect of genetically predicted sarcopenia on the risk of NAFLD using the inverse-variance weighted (IVW) method. Then we performed reverse MR analysis with four SNPs as instruments to examine the causality of genetically predicted NAFLD with whole body lean mass and appendicular lean mass. Further sensitivity analysis was conducted to testify the reliability of the MR results.

**Results:**

Genetic predisposition to decreased whole body lean mass was not associated with NAFLD [IVW-random effects, odds ratio (OR) = 1.054, 95%CI: 0.750–1.482, *P* = 0.761]. Similar results were observed using genetic instruments of appendicular lean mass (IVW-random effects, OR = 0.888, 95%CI: 0.386–2.042, *P* = 0.780). Reverse MR analysis revealed that genetically predicted NAFLD using four genetic instruments was not associated with whole body lean mass (IVW, β = −0.068, 95%CI: −0.179 to 0.043, *P* = 0.229) and appendicular lean mass (IVW, β = −0.020, 95%CI: −0.092 to 0.051, *P* = 0.574). MR analyses using other methods and sensitivity analysis showed consistent results.

**Conclusion:**

These results suggested no causal relationships between sarcopenia and NAFLD, indicating that sarcopenia may not be directly involved in the pathogenesis of NAFLD and vice versa.

## Introduction

Nonalcoholic fatty liver disease (NAFLD) has emerged as the most common chronic liver disease worldwide with a prevalence of about 25% in adult population (1). The spectrum of NAFLD comprises of nonalcoholic fatty liver (NAFL), nonalcoholic steatohepatitis (NASH) and NASH-related end-stage liver diseases such as cirrhosis and hepatocellular carcinoma (HCC). It is considered to be a multisystem disease and hepatic component of metabolic syndrome (2). The pathogenesis of NAFLD has been demonstrated to be multifactorial, in which insulin resistance and genetic predisposition are regarded as two critical driven factors. One of the hallmarks of NAFLD is the interaction between the environment and susceptible polygenic host background which endows varied disease phenotype and influences disease progression (3). Over the past decades, accumulating evidences have established links between NAFLD and extrahepatic diseases such as cardiovascular disease (CVD), and type 2 diabetes mellitus (T2DM) (4, 5). Both epidemiological and mechanical investigations reveal mutual effect of NAFLD on other metabolic disorders suggesting NAFLD could serve as both cause and consequence of extrahepatic metabolic diseases (6, 7).

Sarcopenia is known as a reduction in skeletal muscles, which has been described in not only aging but also a variety of pathological conditions outside the elderly population (8). In recent years, the role of sarcopenia in NAFLD has attracted substantial attentions. A series of researches have shown that sarcopenia is not only closely related to the presence of NAFLD but also an independent risk factor of the advancement and deterioration of the disease (9–11). Similar with NAFLD, sarcopenia is also a result of the interaction between genetic and environmental factors (12). The main characteristic of sarcopenia is loss of lean body mass, notably skeletal muscle, which is commonly measured by dual energy X-ray absorptiometry (DXA) or bioelectrical impedance analysis (BIA). Although an inverse correlation between lean body mass and risk of NAFLD has been observed, the causal relationships between sarcopenia and NAFLD are hardly investigated.

Two-sample MR is an approach utilizing genetic variants as instrumental variables (IVs) for exposure to investigate the causal inference between the exposure and outcome. MR analysis has advantages to overcome the issue of confounding and reverse causality and thus widely applied to assess the causal relationships (13). In the present study, we performed a two-sample bidirectional MR investigation with genome-wide association studies (GWAS) summary data of NAFLD, whole body lean mass and appendicular lean mass to explore the causal relationships.

## Methods

### Study design

**FIGURE 1:**
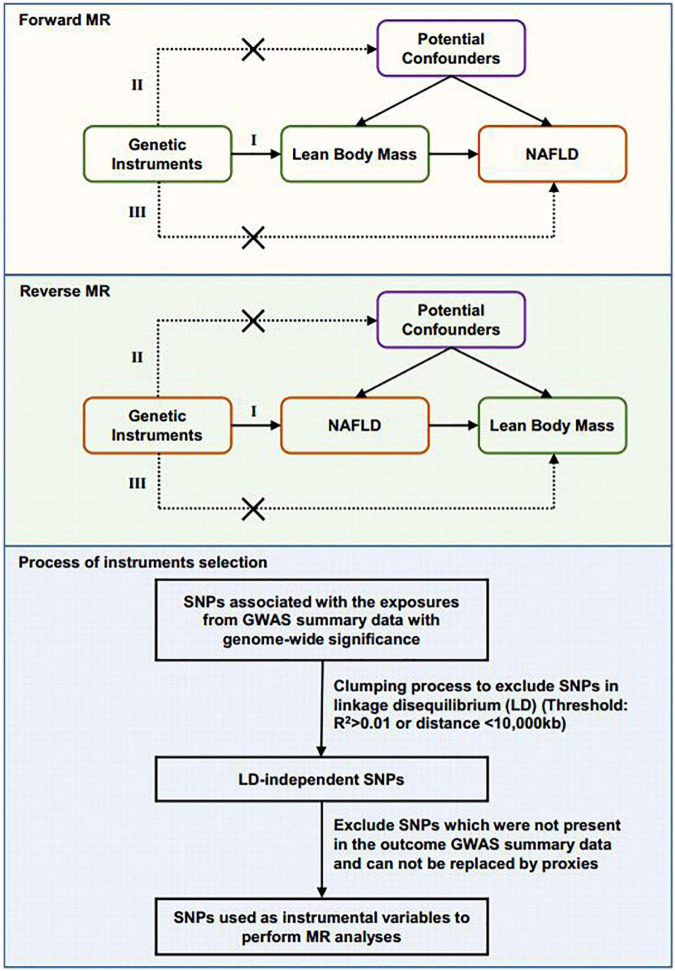
Schematic diagram showing the design of the bidirectional MR analysis and the process of instrument selection. Assumption I: The selected genetic variants are associated with exposure. Assumption II: The genetic instruments are not associated with potential confounders. Assumption III: The genetic instruments are associated with outcome only through the pathway from exposure. The flow chart of genetic instruments selection is shown.

We used a two-sample MR design in which the genetic instruments of exposure and outcome are extracted from independent GWAS data sources. The MR analyses were bidirectional. First, we assessed the causal effect of the whole body lean mass and appendicular lean mass variables on NAFLD. Then we investigated the reverse causal effect of genetically predicted NAFLD on whole body lean mass and appendicular lean mass. The MR analyses conform to three assumptions: (1) genetic variants are associated with the exposure; (2) genetic instruments are not associated with the outcome via confounders; (3) genetic instruments do not affect the outcome directly, only possibly via the exposure (Figure 1). All cited GWAS had been approved by a relevant review board. Ethical approval was not required for this MR study based on summary-level data.

### Data sources for lean body mass

Data regarding the lean body mass were obtained from the up-to-date largest meta-analysis of GWAS, which identified five single nucleotide polymorphisms (SNPs) associated with whole body lean mass in 38,292 individuals of European ancestry and three SNPs associated with appendicular lean mass in 28,330 individuals of European ancestry either genome wide (*P* < 5 × 10^–8^) or suggestively genome wide (*P* < 2.3 × 10^–6^) (14). The identified SNPs were further meta-analyzed and verified in replication cohorts and joint analyses with a total sample size of over 100,000 from 53 studies (14). The lean body mass was measured using DXA or BIA and adjusted for potential confounders such as fat mass, height, sex, age and other study-specific covariates. Then we queried these SNPs in the PhenoScanner database to identify their association with GWAS traits that are potential confounding phenotypes or introduce horizontal pleiotropy. Linkage disequilibrium (defined as *R*^2^ > 0.01 or clump distance < 10,000 kb) between SNPs was assessed based on the 1,000 Genomes European reference panel. Qualified SNPs are selected as instruments after these steps.

### Data source for nonalcoholic fatty liver disease

Summary data of NAFLD were extracted from a large GWAS by Anstee et al. which included 1,483 biopsy-proven NAFLD cases and 17,781 genetically matched controls of European ancestry (15). We estimated the potential bias caused by participants overlap in the data sources of lean body mass and NAFLD which may introduce inflate type I error rate by an online tool.^1^ The results showed low bias risk from sample overlap. Candidate SNPs which met a genome-wide significance threshold (*P* < 5 × 10^–8^) were screened. Linkage disequilibrium was assessed as described above. For SNPs in linkage disequilibrium, those with the strongest association with the exposure were retained. SNPs that were unavailable in the outcome dataset were replaced by suitable proxies (*R*^2^ ≥ 0.8) where available. Palindromic SNPs were also replaced by suitable proxies (*R*^2^ ≥ 0.8). To avoid potential bias from population stratification, we selected proxies using European population reference.

### Statistical analysis

To evaluate the weak instrument bias, we first calculated the F statistics using the formula F = $\left(\frac{N-K-1}{K}\right)\,\left(\frac{R^{2}}{1-R^{2}}\right)$, where N is the sample size, K is the number of IVs, and *R*^2^ is the proportion of the variability of the exposure explained by IVs. And the statistical power was estimated using an online tool (16). We used the inverse variance-weighted (IVW) method under random or fixed effects as the primary statistical method for the bidirectional MR analysis. Cochran’s *Q*-test was calculated to assess the heterogeneity across the individual effect estimates derived from each genetic variant. If significant heterogeneity (*P* < 0.05) was observed, a random-effect IVW model was applied. Other methods we used for MR included Weighted median regression, MR-Egger regression, MR-Pleiotropy RESidual Sum and Outlier (MR-PRESSO) analysis, and Weighted mode to examine the consistency of results and potential pleiotropy. Weighted median method was used to provide a causal estimate assuming more than half of the weight in the analysis comes from valid IVs (17, 18). MR-Egger analysis is applied to detect horizontal pleiotropy by its intercept (*P* < 0.05 for intercept indicates pleiotropy) and generate estimates after correcting for pleiotropic effects (19). MR-PRESSO includes detection of horizontal pleiotropy (MR-PRESSO global test), correction for horizontal pleiotropy via outlier removal (MR-PRESSO outlier test), and testing of significant differences in the causal estimates before and after correction for outliers (MR-PRESSO distortion test). The MR-PRESSO outlier test requires that at least 50% of the variants are valid instruments, has balanced pleiotropy, and relies on the Instrument Strength Independent of Direct Effect (InSIDE) condition that instrument-exposure and pleiotropic effects are uncorrelated. Weighted mode method estimates the causal effect of the subset with the largest number of SNPs by clustering the SNPs into subsets resting on the resemblance of causal effect (20). All analyses were performed using the TwoSampleMR and MR-PRESSO packages in R software (v4.1.3, R Foundation for Statistical Computing, Vienna, Austria) (21, 22).

## Results

### Effect of genetically predicted lean body mass on nonalcoholic fatty liver disease

**FIGURE 2:**
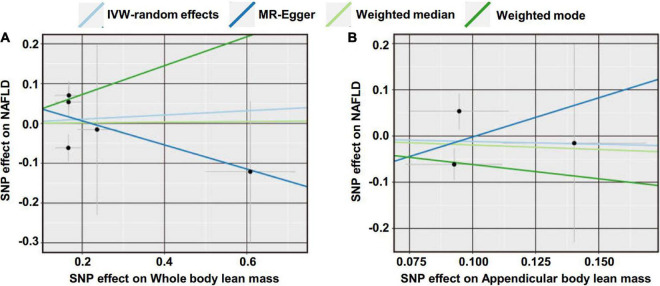
Scatter plot of the causal effect of lean body mass on NAFLD using different MR methods. **(A)** Causal effect of whole body lean mass on NAFLD. **(B)** Causal effect of appendicular lean mass on NAFLD.

**FIGURE 3:**
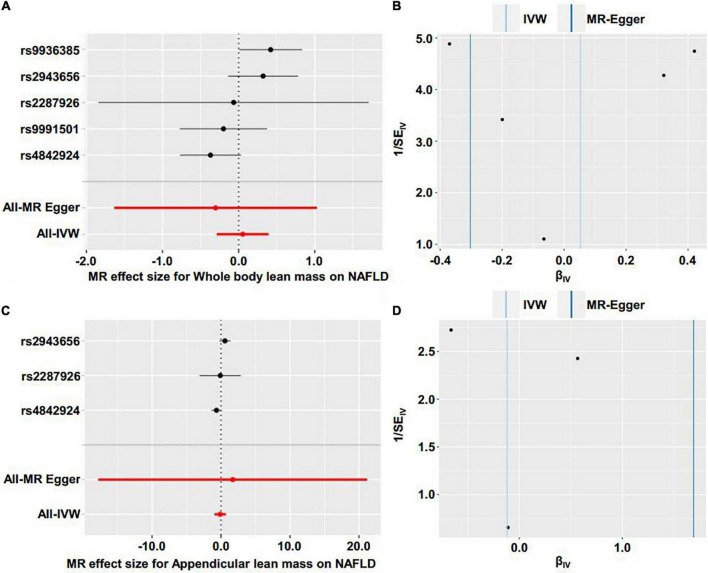
Forest plot and funnel plot of the causal effect of lean body mass on NAFLD. **(A)** Forest plot of the causal effect of whole body lean mass on NAFLD. **(B)** Funnel plot of the causal effect of whole body lean mass on NAFLD. **(C)** Forest plot of the causal effect of appendicular lean mass on NAFLD. **(D)** Funnel plot of the causal effect of appendicular lean mass on NAFLD.

We first investigated the causality of genetically predicted whole body lean mass and appendicular lean mass on NAFLD. For whole body lean mass, five SNPs were selected as IVs. These included rs2943656 in/near IRS1, rs9991501 in/near HSD17B11, rs2287926 in/near VCAN, rs4842924 in/near ADAMTSL3, and rs9936385 in/near FTO which are all available in NAFLD GWAS (Table 1). Forward MR analysis results are shown in Table 2 and Figure 2, which showed no causal effect of whole body lean mass on NAFLD in IVW-random effects method (OR = 1.054, 95%CI: 0.750–1.482, *P* = 0.761). MR analyses in other methods showed consistent results (Table 2 and Figures 2A, 3A,B). Significant heterogeneity was detected (MR-Egger Q = 8.534, *P* = 0.036; IVW Q = 9.379, *P* = 0.052) which suggested the use of random effect model of IVW. No horizontal pleiotropy was observed by intercept estimated from MR-Egger regression (Table 2) and MR-PRESSO global test (*P* = 0.099).

**TABLE 1:** Genetic instruments for lean body mass and their associations with NAFLD.

SNP	Chr	Pos	Gene	EA	OA	Whole body lean mass			NAFLD		
						Beta	SE	*P*	OR	SE	*P*
rs2943656	2	226,830,162	IRS1	A	G	–0.167	0.032	2.536 × 10^–7^	0.948	0.039	0.169
rs9991501	4	88,477,507	HSD17B11	T	C	–0.609	0.110	2.897 × 10^–8^	1.129	0.178	0.495
rs2287926	5	82,851,164	VCAN	A	G	0.236	0.048	8.592 × 10^–7^	0.985	0.214	0.943
rs4842924	15	82,378,611	ADAMTSL3	T	C	–0.166	0.031	1.375 × 10^–7^	0.940	0.034	0.071
rs9936385	16	52,376,670	FTO	T	C	–0.168	0.034	1.115 × 10^–6^	1.073	0.035	0.046

**SNP**	**Chr**	**Pos**	**Gene**	**EA**	**OA**	**Appendicular lean mass**			**NAFLD**		
						**Beta**	**SE**	***P***	**OR**	**SE**	***P***

rs2943656	2	226,830,162	IRS1	A	G	–0.095	0.020	1.141 × 10^–6^	0.948	0.039	0.169
rs2287926	5	82,851,164	VCAN	A	G	0.140	0.029	8.120 × 10^–7^	0.985	0.214	0.943
rs4842924	15	82,378,611	ADAMTSL3	T	C	–0.093	0.019	1.232 × 10^–6^	0.940	0.034	0.071

SNP, single nucleotide polymorphism; Chr, chromosome; Pos, position based on hg19; EA, effect allele; OA, other allele; NAFLD, nonalcoholic fatty liver disease; OR, odds ratio.

**TABLE 2:** MR estimates from different methods assessing the causal effect of lean body mass on NAFLD.

Exposure	Outcome	Method	OR	95%CI	*P*
Whole body lean mass (5 SNPs, *F* statistic = 26.682, MR power = 0.05)	NAFLD	IVW-random effects	1.054	0.750–1.482	0.761
		Weighted median	1.008	0.741–1.371	0.961
		MR-Egger	0.739	0.195–2.799	0.686
		Cochran’s *Q* = 8.533 (*P* = 0.036); MR-Egger intercept = 0.067 (*P* = 0.624)			
		MR-PRESSO	1.054	0.750–1.482	0.776
		Weighted mode	1.437	0.813–2.541	0.280
Appendicular lean mass (3 SNPs, *F* statistic = 23.830, MR power = 0.06)	NAFLD	IVW-random effects	0.888	0.386–2.042	0.780
		Weighted median	0.823	0.389–1.739	0.610
		MR-Egger	5.432	1.866 × 10^–8^–1.581 × 10^9^	0.893
		Cochran’s *Q* = 4.801 (*P* = 0.028); MR-Egger intercept = −0.171 (*P* = 0.885)			
		Weighted mode	0.540	0.217–1.346	0.317

OR, odds ratio; CI, confidence interval; SNP, single nucleotide polymorphism; NAFLD, nonalcoholic fatty liver disease; IVW, inverse variance weighted; PRESSO, pleiotropy residual sum and outlier.

For appendicular lean mass, rs2943656, rs2287926, and rs4842924 were selected as IVs to conduct the forward MR analysis. Similarly, no evidence supporting the causal effect of appendicular lean mass on NAFLD was obtained in IVW-random effects method (OR = 0.888, 95%CI: 0.386–2.042, *P* = 0.780) and in other MR methods (Table 2 and Figures 2B, 3C,D). The heterogeneity test showed significant heterogeneity in MR-Egger Cochran’s *Q*-test (*Q* = 4.801, *P* = 0.028) and borderline significant heterogeneity of estimates derived from the three SNPs in IVW Cochran’s *Q*-test (*Q* = 4.961, *P* = 0.084). Horizontal pleiotropy test showed no significant results by MR-Egger intercept (Table 2). MR-PRESSO global test was unable to be conducted due to the lack of enough IVs.

### Effect of genetically predicted nonalcoholic fatty liver disease on lean body mass

**FIGURE 4:**
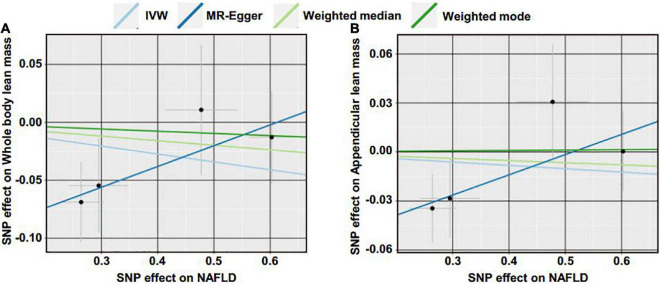
Scatter plot of the causal effect of NAFLD on lean body mass using different MR methods. **(A)** Causal effect of NAFLD on whole body lean mass. **(B)** Causal effect of NAFLD on appendicular lean mass.

**FIGURE 5:**
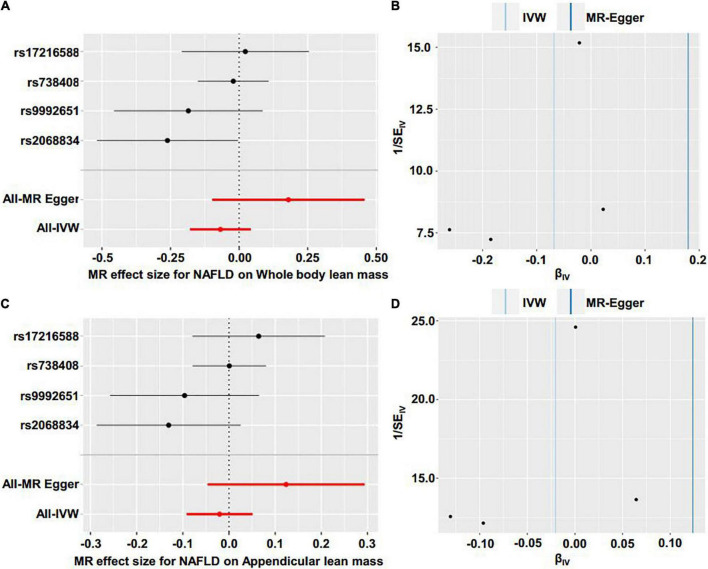
Forest plot and funnel plot of the causal effect of NAFLD on lean body mass. **(A)** Forest plot of the causal effect of NAFLD on whole body lean mass. **(B)** Funnel plot of the causal effect of NAFLD on whole body lean mas. **(C)** Forest plot of the causal effect of NAFLD on appendicular lean mass. **(D)** Funnel plot of the causal effect of NAFLD on appendicular lean mass.

Reverse MR analyses were then conducted to assess the causal effect of NAFLD on lean body mass. Four SNPs including rs2068834 in/near ZNF512, rs9992651 in/near HSD17B13, rs17216588 in/near CLIP2, and rs738408 in/near PNPLA3 were utilized as IVs of NAFLD, in which rs738408 was used as a proxy for rs738409 in the MR analyses as the latter is a palindromic SNP (Table 3). MR analysis by IVW method showed that there was no causal effect of NAFLD on whole body lean mass (β = −0.068, 95%CI: −0.179 to 0.043, *P* = 0.229). Consistent results were obtained using other MR methods (Table 4 and Figures 4A, 5A,B). Heterogeneity analysis showed no significant results (MR-Egger Q = 0.499, *P* = 0.779; IVW Q = 3.975, *P* = 0.264). Moreover, there was no horizontal pleiotropy tested by intercept estimated from MR-Egger regression (Table 4) and MR-PRESSO global test (*P* = 0.376).

**TABLE 3:** Genetic instruments for NAFLD and their associations with lean body mass.

SNP	Chr	Pos	Gene	EA	OA	NAFLD			Whole body lean mass		
						OR	SE	*P*	Beta	SE	*P*
rs2068834	2	27839539	ZNF512	T	C	1.302	0.041	8.486 × 10^–11^	0.069	0.035	0.046
rs9992651	4	88232510	HSD17B13	A	G	0.744	0.053	2.769 × 10^–8^	0.055	0.041	0.182
rs17216588	19	19664077	CILP2	T	C	1.612	0.064	7.245 × 10^–14^	0.011	0.057	0.849
rs738408	22	44324730	PNPLA3	T	C	1.827	0.041	1.591 × 10^–49^	−0.013	0.040	0.748

**SNP**	**Chr**	**Pos**	**Gene**	**EA**	**OA**	**NAFLD**			**Appendicular lean mass**		
						**OR**	**SE**	***P***	**Beta**	**SE**	***P***

rs2068834	2	27839539	ZNF512	C	T	1.302	0.041	8.486 × 10^–11^	0.035	0.021	0.099
rs9992651	4	88232510	HSD17B13	A	G	0.744	0.053	2.769 × 10^–8^	0.028	0.024	0.242
rs17216588	19	19664077	CILP2	T	C	1.612	0.064	7.245 × 10^–14^	0.031	0.035	0.380
rs738408	22	44324730	PNPLA3	T	C	1.827	0.041	1.591 × 10^–49^	0.000	0.025	0.987

SNP, single nucleotide polymorphism; Chr, chromosome; Pos, position based on hg19; EA, effect allele; OA, other allele; NAFLD, nonalcoholic fatty liver disease; OR, odds ratio.

**TABLE 4:** MR estimates from different methods assessing the causal effect of NAFLD on lean body mass.

Exposure	Outcome	Method	Beta	95%CI	*P*
NAFLD (4 SNPs, F statistic = 74.299, MR power = 0.43)	Whole body lean mass	IVW	–0.068	−0.179 to 0.043	0.229
		Weighted median	–0.040	−0.150 to 0.071	0.484
		MR-Egger	0.180	-0.098 to 0.457	0.333
		Cochran’s *Q* = 0.499 (*P* = 0.779); MR-Egger intercept = −0.110 (*P* = 0.203)			
		MR-PRESSO	–0.068	−0.179 to 0.043	0.315
		Weighted mode	–0.019	−0.142 to 0.104	0.780
NAFLD (4 SNPs, F statistic = 74.299, MR power = 0.08)	Appendicular lean mass	IVW	–0.020	−0.092 to 0.051	0.574
		Weighted median	–0.013	−0.082 to 0.056	0.709
		MR-Egger	0.124	-0.046 to 0.294	0.290
		Cochran’s *Q* = 1.230 (*P* = 0.541); MR-Egger intercept = −0.063 (*P* = 0.218)			
		MR-PRESSO	–0.020	−0.092 to 0.051	0.613
		Weighted mode	0.002	−0.076 to 0.081	0.955

OR, odds ratio; CI, confidence interval; NAFLD, nonalcoholic fatty liver disease; SNP, single nucleotide polymorphism; IVW, inverse variance weighted; PRESSO, pleiotropy residual sum and outlier.

Next, we examined the causal effect of genetically predicted NAFLD on appendicular lean mass. No evidence was shown supporting the causality by IVW method (β = −0.020, 95%CI: −0.092 to 0.051, *P* = 0.574) and by other methods (Table 4 and Figures 4B, 5C,D). There were no significant heterogeneity (MR-Egger Q = 1.230, *P* = 0.541; IVW Q = 4.374, *P* = 0.224) and horizontal pleiotropy (MR-Egger intercept = −0.063, *P* = 0.218; MR-PRESSO global test *P* = 0.324) in this section.

## Discussion

Although a close link between sarcopenia and NAFLD has been established, the causal relationships between the two pathological conditions are not examined. Here, we first investigated the causality by a two-sample bidirectional MR approach and discovered no causal relationships between lean body mass and NAFLD.

Recent studies have identified the association of sarcopenia with NAFLD and NAFLD-related advanced fibrosis, independent of insulin resistance and obesity (9–11, 23). Furthermore, sarcopenia has been shown be a risk factor of poor outcomes in NAFLD and other chronic liver diseases (24–26). However, whether sarcopenia directly contributes to NAFLD or vice versa is not yet clarified. Indeed, mechanistic studies may provide more insights into the direct link between the two pathological conditions. Our analyses provided evidences from genetic perspectives using the MR approach which diminishing reverse causality and minimizing residual confounding. The results of our study seemed to be inconsistent with the clinical observations mentioned above, in which no causal link between sarcopenia and NAFLD was established. A variety of reasons may underlie the inconsistency. Firstly, the association between sarcopenia and NAFLD observed by the cross-sectional studies may be mediated by potential confounders. Although some confounding factors such as obesity, T2DM and other components of metabolic syndrome may have been adjusted in some investigations, potential confounders may still exist. In fact, substantial factors have been shown to be involved in both sarcopenia and NAFLD and are considered to be shared pathological foundations. For instance, insulin resistance has been demonstrated to be correlated with both sarcopenia and NAFLD (27, 28). Insulin resistance promotes glycogenesis, accelerates protein degradation, reduces protein synthesis and induces myostatin which lead to the decline of skeletal muscle. In turn, the loss of skeletal muscle exacerbates insulin resistance as skeletal muscle accounts for about 80% of glucose disposal (29, 30). Similarly, insulin resistance is able to initiate and facilitate the progression of NAFLD by multiple pathways including promotion of hepatic *de novo* lipogenesis, activation of adipose tissue lipolysis and so on (31). And NAFLD also contributes to insulin resistance by combined mechanisms such as increasing lipid metabolites including ceramides and diacylglycerols which inhibit insulin signaling (31). Due to the fact that insulin resistance interferes with both sarcopenia and NAFLD, it can mediate the establishment of association between the two conditions without causal relationships. Furthermore, genetically determined NAFLD may differ from metabolically determined NAFLD in the pathogenic basis. It has been found that variants in PNPLA3 is strongly associated with NAFLD in the absence of insulin resistance or dyslipidemia (32, 33). Therefore, the association between genetically determined NAFLD and sarcopenia need further explorations.

Moreover, most of the coexistence of sarcopenia and NAFLD were studied in patients with advanced stage of NAFLD, which cannot reflect sarcopenia’s contribution to the susceptibility of NAFLD. Intriguingly, a recent prospective investigation revealed that myosteatosis but not sarcopenia predisposes early stage NAFLD patients to early NASH and fibrosis progression, suggesting myosteatosis may play a more important role in the development and progression of early stage NAFLD (34). Therefore, the role of sarcopenia and myosteatosis in the pathogenesis of NAFLD should be further determined by more evidences from clinical and mechanistic studies. Our results also showed that there was no causal effect of lean body mass on NAFLD, which implied that sarcopenia may not contribute to the predisposition of NAFLD. More importantly, most of the clinical investigations on the association of sarcopenia with NAFLD were conducted in Asian countries while our MR analyses used data sources from GWAS studies in European ancestry, which may cause discrepancy of the results between our MR analyses and clinical studies in Asian populations. Therefore, large-scale GWAS studies on sarcopenia and NAFLD in Asian populations are needed to further examine the causal relationships between sarcopenia and NAFLD in Asian ancestry. Moreover, the SNPs we chose to genetically predict NAFLD were limited by the GWAS summary data we utilized for the MR analysis. More GWAS studies with large sample size would help to identify SNPs to better genetically predict NAFLD and facilitate examining our results with other proxy SNPs. Notably, a recent investigation have made further analyses based on the former GWAS data and discovered that the SNPs of lean body mass may have different and even contradictory effects on the metabolic phenotypes (35). Therefore, further GWAS analyses on the genetic characteristics of lean body mass are needed to identify the intrinsic link with metabolic phenotype and provide more suitable SNP candidates to conduct MR investigations on the causal effect of lean body mass on metabolic disorders including NAFLD.

The major finding of the present MR study is that there are no causal relationships between genetically predicted lean body mass and NAFLD. These results indicate that sarcopenia and NAFLD may not share common genetic background although certain metabolic disorders such as insulin resistance and adiposity are supposed to be involved in both pathological conditions. Therefore, the link between sarcopenia and NAFLD is likely to be established by modifiable factors that can be subjected to clinical interventions. There are some limitations that should be considered when interpreting the results of the present MR study. Firstly, the statistical power of the MR analyses were relatively low due to the limited sample size which implies that further MR studies based on larger scale GWAS of sarcopenia and NAFLD are needed to verify the causality. Secondly, epidemiological studies have shown that the risks of both sarcopenia and NAFLD are affected by gender and age (36–39). However, we could not assess the causality between genetically predicted sarcopenia and NAFLD in different genders and age groups due to the lack of gender and age-stratified data. Thirdly, as we have mentioned above, the effects of SNPs on the traits may be inconsistent or even contradictory which makes it hard to select suitable instruments that serve as true proxies for exposures. Therefore, function investigations of the SNPs may be needed to help interpret the genetic characteristics of sarcopenia and NAFLD and facilitate more precise selection of instruments.

In summary, the present two-sample bidirectional MR study suggested that there are no causal relationships between sarcopenia and NAFLD. The results provide novel insights into the pathogenesis of sarcopenia and NAFLD from genetic perspectives and further studies based on larger scale GWAS are needed to verify the conclusions.

## Data availability statement

The original contributions presented in this study are included in the article/Supplementary material, further inquiries can be directed to the corresponding author.

## Author contributions

Z-HZ, Y-CF, and KW contributed to the study conception, design, and manuscript drafting. Z-HZ, JZ, and XH contributed to the acquisition and analysis of data. All authors approved the final manuscript.

## Footnotes

1
https://sb452.shinyapps.io/overlap/
