# Supplementary material for: Assessing causal relationships between sarcopenia and nonalcoholic fatty liver disease: A bidirectional Mendelian randomization study
Source: Front Nutr. 2022 Nov 9;9:971913. doi: 10.3389/fnut.2022.971913 (PMC9682105; doi:10.3389/fnut.2022.971913)
Supplement: Supplementary file 1 [file Data_Sheet_1.DOCX]

**Supplementary Information**

**Assessing Causal Relationships Between Sarcopenia and Nonalcoholic Fatty Liver Disease: A Bidirectional Mendelian Randomization Study**

Ze-Hua Zhao^1^, Juanjuan Zou^2^, Xin Huang^3^, Yu-Chen Fan^1,4^, Kai Wang^1,4*^

^1^Department of Hepatology, Qilu Hospital of Shandong University, Jinan 250012, China; ^2^Department of Otorhinolaryngology, Qilu Hospital of Shandong University, NHC Key Laboratory of Otorhinolaryngology (Shandong University), Jinan 250012, China; ^3^Division of Bariatric and Metabolic Surgery, Department of General Surgery, Qilu Hospital of Shandong University, Jinan 250012, China; ^4^Institute of Hepatology, Shandong University, Jinan 250012, China

**Content**

Supplementary Table 1

Supplementary Figure Legends

**Supplementary Table 1. Summary information of the selected GWAS studies.**

| Exposure/Outcome | PMID | Population | Sample size | Consortium |
| --- | --- | --- | --- | --- |
| NAFLD | 32298765 | European | 1483 cases and 17,781 controls | NA |
| Whole body lean mass | 28724990 | European | 38,292 | GEFOS |
| Appendicular lean mass | 28724990 | European | 28,330 | GEFOS |

NAFLD, nonalcoholic fatty liver disease; NA, not available; GEFOS, GEnetic Factors for Osteoporosis

**Supplementary Figure Legends**

**Supplementary Figure 1. Forest plots with the method of leave-one-out.** (A) Forest plot with the method of leave-one-out for the causal effect of whole body lean mass on NAFLD. (B) Forest plot with the method of leave-one-out for the causal effect of appendicular lean mass on NAFLD. (C) Forest plot with the method of leave-one-out for the causal effect of NAFLD on whole body lean mass. (D) Forest plot with the method of leave-one-out for the causal effect of NAFLD on appendicular lean mass.
