# Supplementary figures and images for: Assessing causal relationships between sarcopenia and nonalcoholic fatty liver disease: A bidirectional Mendelian randomization study
Source: Front Nutr. 2022 Nov 9;9:971913. doi: 10.3389/fnut.2022.971913 (PMC9682105; doi:10.3389/fnut.2022.971913)

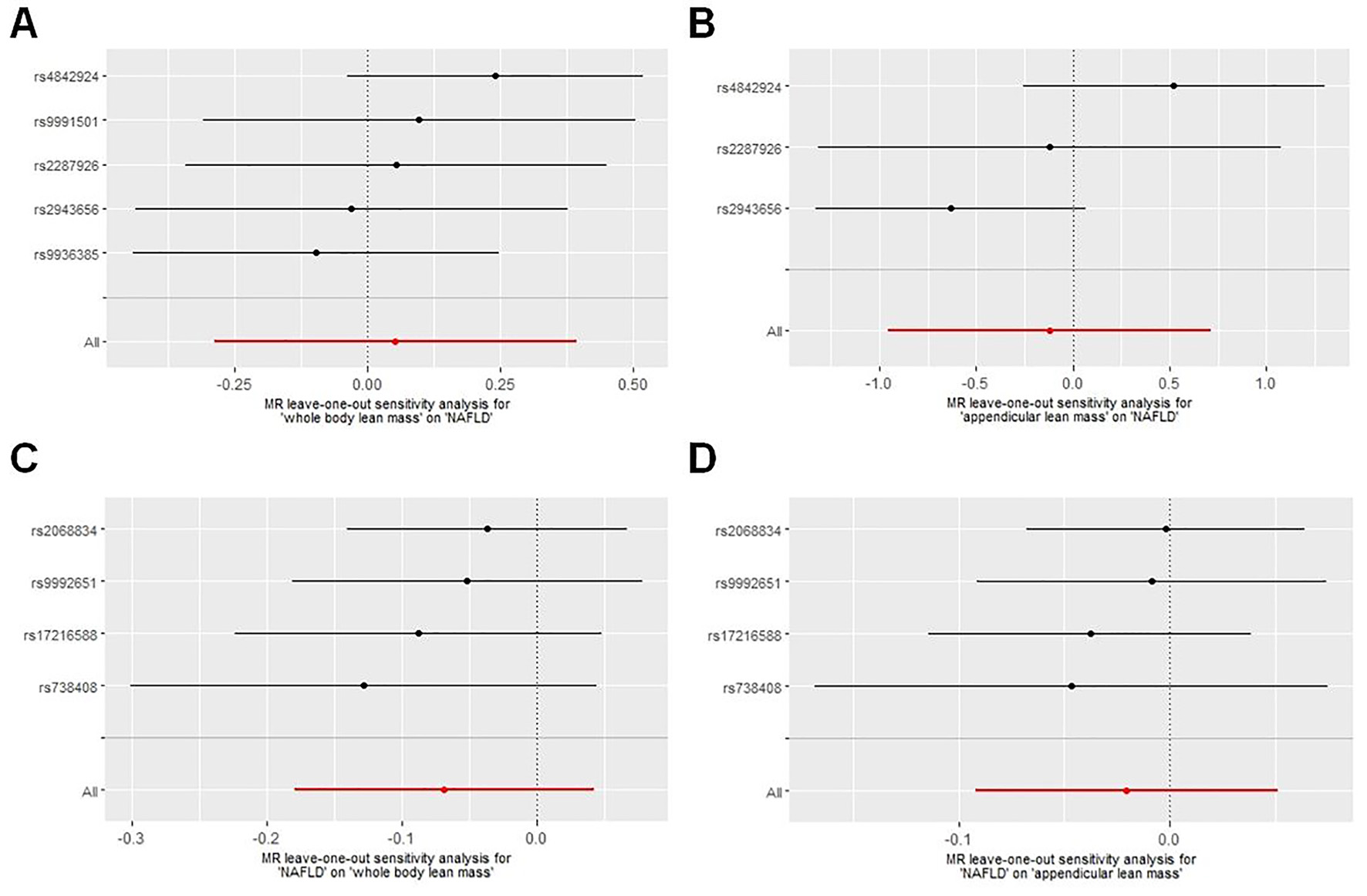

Supplement: Supplementary file 2 [file Image_1.JPEG]
